# Reluctance to Video Recording in Functional Movement Disorders: A Cross‐Sectional Study

**DOI:** 10.1002/brb3.71638

**Published:** 2026-07-28

**Authors:** Daniela Kern, Robert Matic, Lukas Gattermeyer‐Kell, Sebastian Franthal, Mariella Kögl, Petra Katschnig‐Winter, Maria Schneller, Christian Enzinger, Petra Schwingenschuh

**Affiliations:** ^1^ Department of Neurology Medical University of Graz Graz Austria

**Keywords:** dystonia, functional neurological disorder, movement disorders, tremor

## Abstract

**Background:**

Video recording is an important tool for education in movement disorders, particularly in functional movement disorders (FMD). In this study, we examined factors associated with patients’ consent to video recording during the neurological examination in individuals with functional and other movement disorders.

**Methods:**

In this cross‐sectional study, patients with FMD (*n* = 42), essential tremor (ET) (*n* = 18), isolated dystonia (*n* = 34), and Parkinson's disease (PD) (*n* = 34) underwent a standardized neurological examination. Patients were asked to consent to video recording of the examination. The primary outcome was consent to video recording (yes/no). Health‐related quality of life (HRQoL), anxiety, depression, and personality traits were assessed using questionnaires.

**Results:**

Of the 128 included patients, 78.9% consented to video recording. Patients with FMD were more likely to refuse video recording than patients with non‐FMDs (OR = 4.20, 95% CI (1.73–10.20), *p* = 0.002). Female sex was associated with a lower likelihood of consenting to be filmed (OR = 0.34, 95% CI (0.12–0.97), *p* = 0.044). No differences in HRQoL, anxiety, depression, or personality traits were found between patients who declined and those who consented to be filmed.

**Conclusions:**

Patients with FMD were more reluctant to be filmed during the neurological examination than patients with non‐FMDs. This difference cannot be explained by sex, comorbidities, or personality traits alone but instead may reflect factors specific to FMD.

## 1 Introduction

Functional movement disorders (FMD) are disabling motor symptoms experienced as involuntary and diagnosed on the basis of internal inconsistency (Gupta and Lang 2009). The associated disability is comparable to other chronic neurological conditions (Carson et al. 2011). Yet, diagnostic delay remains common despite increasing awareness and improved diagnostic criteria (Butler et al. 2021). Persistent reluctance to diagnose FMD (McLoughlin et al. 2025) and repeatedly reported gaps in education (De Liège et al. 2022) underscore the importance of high‐quality teaching material for clinicians in training.

Video recordings are central to education in movement disorders and are particularly valuable in FMD for demonstrating positive diagnostic signs. Their use for educational and scientific purposes depends on patients’ consent, yet factors influencing this willingness remain poorly understood. We therefore aimed to identify clinical and psychological factors associated with consent to video recording in patients with functional and other movement disorders.

## 2 Materials and Methods

We conducted a cross‐sectional study of 128 consecutive patients attending the Movement Disorders Outpatient Clinic at the Medical University of Graz between January 2023 and July 2025. Patients were aged 18–80 years and diagnosed with FMD, essential tremor (ET), isolated dystonia, or Parkinson's disease (PD) according to established criteria (Albanese et al. 2025, Bhatia et al. 2018, Postuma et al. 2015). Patients with diagnosed dementia were excluded. All participants provided written informed consent.

Each patient underwent a standardized neurological examination. Disease severity was assessed using either the simplified FMDs Rating Scale (S‐FMDRS), the Fahn‐Tolosa‐Marin Tremor Rating Scale (FTM), the Toronto Western Spasmodic Torticollis Rating Scale (TWSTRS), or the Movement Disorders Society‐Unified PD Rating Scale (MDS–UPDRS), as appropriate.

Patients were asked to consent to video recording of the examination and to specify whether recordings could be used exclusively for clinical purposes (e.g., documenting disease progression), or also for education or publication. This was documented on a standardized consent‐form. The primary outcome was consent to video recording (yes/no).

Participants completed questionnaires assessing health‐related quality of life (HRQoL), anxiety, depression, and personality styles, including the EuroQol five‐dimension five‐level questionnaire (EQ‐5D‐5L), General Anxiety Disorder‐7 (GAD‐7), the Patient Health Questionnaire‐9 (PHQ‐9), and the Personality Inventory for DSM‐5 Brief Form (PID‐5‐BF).

The study was approved by the ethics committee (34‐509ex21/22) and conducted in accordance with the Declaration of Helsinki.

Statistical analysis was performed using IBM SPSS Statistics, version 29.0. Categorical variables were analyzed using chi‐square or Fisher's exact‐test, and continuous variables with one‐way ANOVA. To examine the association between diagnostic groups and video recording refusal, binary logistic regression analyses were performed using the forced entry method (Enter). Predictors (diagnosis and sex) were entered simultaneously into the model to calculate adjusted odds ratios and the corresponding 95% confidence intervals. Post hoc pairwise comparisons were performed using the Bonferroni correction when homogeneity of variance was met and the Games‐Howell tests when this assumption was violated. The significance of individual model predictors was evaluated using the Wald statistic. *P*‐values < 0.05 were considered significant. Artificial intelligence (ChatGPT, OpenAI) was used to assist with language editing and to improve the readability of the manuscript.

## 3 Results

A total of 128 patients were included: 42 with FMD, 18 with ET, 34 with dystonia, and 34 with PD. Overall, 78.9% consented to video recording, while 21.1% declined. Table 1 summarizes demographic and clinical characteristics. Mean disease rating scales were 9 (SD 5) for the S‐FMDRS in FMD, 30 (SD 16) for the FTM in ET, 17 (SD 10) for the (TWSTRS in dystonia, and 31 (SD 13) for the MDS–UPDRS in patients with PD. FMD phenomenology comprised functional tremor (*n* = 23), mixed FMD (*n* = 11), functional dystonia (*n* = 4), functional paroxysmal movement disorder (*n* = 2), functional gait disorder (*n* = 1), and functional myoclonus (*n* = 1).

**TABLE 1:** Demographic, clinical, and psychological characteristics.

**A. Demographic and clinical characteristics**.						
—	FMD (*n* = 42)	ET (*n* = 18)	ID (*n* = 34)	PD (*n* = 34)	—	—
Female, *n* (%)	22 (52.4)	9 (50)	28 (82.4)	8 (23.5)	—	—
Male, *n* (%)	20 (47.6)	9 (50)	6 (17.6)	26 (76.5)	—	—
Age in years, mean (SD) (range)	46 (14) (18–69)	58 (18) (22–78)	60 (12) (35–77)	62 (10) (32–75)	—	—
Disease duration in years, mean (SD) (range)	7 (7) (0–25)	21 (18) (3–55)	16 (12) (1–44)	7 (5) (1–18)	—	—
BMI, mean (SD)	25.7 (4.8)	25.5 (4.6)	25.0 (4.8)	27.6 (4.8)	—	—
Consent to video recording, n (%)	26 (61.9)	17 (94.4)	26 (76.5)	32 (94.1)	—	—
No consent to video recording (%)	16 (38.1)	1 (5.6)	8 (23.5)	2 (5.9)	—	—
**B. Quality of life and psychological characteristics**.						
—	FMD (*n* = 42)	ET (*n* = 18)	ID (*n* = 34)	PD (*n* = 34)	ANOVA	Post hoc
EQ‐5D‐5L utility scores, mean (SD)	0.64 (0.36)	0.87 (0.19)	0.88 (0.13)	0.84 (0.20)	*F(3, 56.17) = 5.18, *p* = 0.003, *ƞ* ^2^ = 0.15	FMD < PD (*p* = 0.023) FMD < ID (*p* = 0.001) FMD < ET (*p* = 0.011)
GAD‐7, mean (SD)	7 (5)	3 (2)	6 (5)	5 (5)	F(3, 101) = 2.81, *p* = 0.043, *ƞ* ^2^ = 0.08	FMD > ET (*p* = 0.050)
PHQ‐9, mean (SD)	9 (7)	4 (3)	7 (5)	7 (6)	*F(3, 49.29) = 3.91, *p* = 0.014, *ƞ* ^2^ = 0.07	FMD > ET (*p* = 0.010)
Negative affectivity (PID‐5‐BF), mean (SD)	2 (1.3)	1.3 (0.9)	1.9 (1.3)	1.7 (1.2)	*F*(3, 98) = 0.98, *p* = 0.405, *ƞ* ^2^ = 0.03	—
Detachment (PID‐5‐BF), mean (SD)	1.3 (1.1)	1.3 (1.0)	1.2 (1.0)	1.5 (0.9)	*F*(3, 98) = 0.42, *p* = 0.737, *ƞ* ^2^ = 0.01	—
Antagonsim (PID‐5‐BF), mean (SD)	0.8 (0.8)	0.7 (0.6)	0.7 (0.8)	1.0 (0.8)	*F*(3, 98) = 0.75, *p* = 0.528, *ƞ* ^2^ = 0.02	—
Disinhibition (PID‐5‐BF), mean (SD)	1.5 (1.2)	1.7 (1.1)	1.3 (0.9)	1.3 (1.0)	*F*(3, 98) = 0.67, *p* = 0.572, *ƞ* ^2^ = 0.02	—
Anankastia (PID‐5‐BF), mean (SD)	2.3 (1.6)	1.7 (1.0)	1.8 (0.9)	2.1 (1.1)	*F(3, 43.92) = 1.32, *p* = 0.280, *ƞ* ^2^ = 0.04	—
Psychoticism (PID‐5‐BF), mean (SD)	1.6 (1.2)	1.4 (1.0)	1.0 (0.9)	1.4 (1.0)	*F*(3, 98) = 1.67, *p* = 0.178, *ƞ* ^2^ = 0.05	—

Demographic, clinical and psychological characteristics in patients with functional movement disorders (FMD), essential tremor (ET), isolated dystonia (ID), and Parkinson's disease (PD).

Abbreviations: BMI, body mass index; sFMDRS, simplified Functional Movement Disorders Rating Scale; FTM, Fahn‐Tolosa‐Marin Tremor Rating Scale; TWSTRS, Toronto Western Spasmodic Torticollis Rating Scale; MDS‐UPDRS, Movement Disorder Society‐Unified Parkinson's Disease Rating Scale; n.a., not applicable; EQ‐5D‐5L, EuroQol five‐dimension five‐level questionnaire; GAD‐7, Generalized Anxiety Disorder‐7; PHQ‐9, Patient Health Questionnaire‐9; PID‐5‐BF, Personality Inventory for DSM‐5 Brief Form.

*Note*: *Welch‐ANOVA, due to violated homogeneity of variances.

HRQoL varied by diagnosis, with patients with FMD reporting lower HRQoL than patients in all other groups. A one‐way ANOVA showed a significant difference in EQ‐5D‐5L‐utility‐scores between diagnostic groups, *F*(3, 56.17) = 5.18, *p* = 0.003, *ƞ* ^2^ = 0.15, indicating a large effect. They also had higher anxiety‐scores (GAD‐7) than patients with ET, *F*(3, 101) = 2.81, *p* = 0.043, *ƞ* ^2^ = 0.08, representing a medium effect. Similarly, depression‐scores (PHQ‐9) were higher in patients with FMD than patients with ET, *F*(3, 49.29) = 3.91, *p* = 0.014, *ƞ* ^2^ = 0.07, also indicating a medium effect. Personality traits (PID‐5‐BF) were comparable across groups (see Table 1).

Logistic regression analysis showed that FMD patients were more likely to refuse video recording than patients with non‐FMDs (OR = 4.20, 95% CI (1.73–10.20), *p* = 0.002). Compared with individual groups, FMD patients were significantly more likely to decline filming than PD (OR = 9.85, 95% CI (2.07–46.78), *p* = 0.004) and ET patients (OR = 10.46, 95% CI (1.27–86.35), *p* = 0.029), but not compared to dystonia patients (*p* = 0.178).

After adjusting for sex, FMD remained associated with refusal to be recorded compared with PD (adjusted OR = 7.70, 95% CI (1.58–37.66), *p* = 0.012) and ET (adjusted OR 11.16, 95% CI (1.32–94.40), *p* = 0.026). No significant difference was observed between patients with FMD and dystonia (*p* = 0.062). Sex was a predictor of consent to filming, with women being more likely to refuse being filmed than men (OR = 0.34, 95% CI (0.12–0.97), *p* = 0.044).

Patients who refused to be filmed were more frequently diagnosed with an anxiety disorder than those who consented, although this marginally did not reach statistical significance (25.9% vs. 9.9%; *p* = 0.050). The prevalence of depression did not differ significantly between groups (40.7% vs. 27.7%).

Patients who refused and those who gave consent to be filmed did not differ in age, BMI, or years of education (all *p* > 0.050). Likewise, no difference was found in anxiety or depression scores, personality styles, or HRQoL (all *p* > 0.050).

Due to the limited sample size, comparison of disease severity was only feasible in patients with FMD and dystonia. Disease severity in FMD and dystonia did not differ between patients who refused and those who consented to video recording (all *p* > 0.050).

## 4 Discussion

Video recordings are central to education and research in FMD, yet their use depends on patients’ consent, and little is known about clinical and psychological factors influencing patients’ willingness to be filmed. This was addressed in this cross‐sectional study of 128 patients with functional and non‐FMDs. Our findings show that an FMD diagnosis and female sex were associated with refusal to consent to video recording during the neurological examination, independent of disease severity and common psychiatric comorbidities. Patients with FMD were significantly more likely to decline filming than those with PD and ET, whereas differences with dystonia did not reach statistical significance. The association between FMD and refusal to be filmed remained significant after adjusting for sex, suggesting that FMD‐specific factors contribute to this reluctance.

In contrast to a general practice study from 1997, where female patients and those with an overt presentation of psychological disorders were found to be more likely to decline video recording (Howe 1997), our study did not find any association between the willingness to consent to filming and measures of anxiety, depression, personality styles, or HRQoL. In the earlier study, 35% of participants declined video recording. A direct comparison between both studies, however, is not reasonable, as the general practice study was conducted nearly 30 years ago, and video recording has since become more common both in clinical practice and in everyday life.

A literature review from 2016 examined the acceptability of video recording for clinical purposes and reported a mean recruitment rate of 68.2% and a mean retention rate of 73.3% across the included studies. Privacy concerns and self‐consciousness about physical appearance were reported but not investigated systematically. To our knowledge, no previous study has specifically examined the acceptance of video recordings among patients with movement disorders (Lear et al. 2023).

In our study, the lower consent rate among patients with FMD cannot be explained by female sex, psychological comorbidities, or personality related factors. Instead, we suggest that other mechanisms specific to FMD may play a role.

Compared with other chronic neurological conditions like PD, multiple sclerosis, or epilepsy patients with functional neurological disorder (FND) more frequently report high levels of stigma (McLoughlin et al. 2024), which has been conceptualized as labeling, stereotyping, separation, and discrimination (Link and Phelan 2001). Based on a description of stigma in FND by McLoughlin et al., stigma associated with the symptom experience may discourage patients from being filmed. The variability and inconsistency of the symptoms—core features of FND—may feel unusual, embarrassing, or difficult to explain to others (McLoughlin et al. 2024).

Many patients also describe persistent doubts about the legitimacy of their symptoms and fears of being accused of feigning, which could make them particularly sensitive to any situation that might be perceived as evaluative or judgmental, such as video recording for teaching (Lehn et al. 2019).

Interestingly, refusal rates in FMD did not differ significantly from those in dystonia, in contrast to PD and ET. This may reflect partially overlapping psychosocial, clinical, and neurophysiological features between FMD and dystonia, including visible, sometimes socially conspicuous abnormal movements and a high proportion of female patients (LaHue et al. 2020). These factors may similarly increase concerns about being observed and judged on video. Dystonia is also often associated with pain, embarrassment about abnormal postures, and long diagnostic delays (LaHue et al. 2020), all of which may contribute to a reluctance to be filmed that resembles patterns seen in FMD. The lack of a statistically significant difference between FMD and dystonia should therefore not be interpreted as evidence against FMD‐specific mechanisms but rather as suggesting that some of these mechanisms may also be shared with other stigmatizing movement disorders. Furthermore, similar local field potentials in the internal globus pallidus and thalamus have been reported in functional and non‐functional dystonia, suggesting that the two conditions share at least some pathophysiological mechanisms. (Baizabal‐Carvallo et al. 2019)

Finally, for some patients, accepting the FMD diagnosis can be challenging and may conflict with their self‐concept. Thus, having symptoms recorded and shared may render the diagnosis more concrete and therefore harder to ignore or re‐interpret (Bazydlo and Eccles 2024).

These considerations remain speculative, as we did not directly assess stigma or patients’ reasons for refusing video recording. Nonetheless, our findings point to a clinically relevant barrier to generating high‐quality educational material in FMD and highlight the importance of addressing patients’ concerns about how video material will be used and by whom it will be seen.

Limitations of this study include its single‐center design, modest sample size, and the absence of stigma‐specific or consent‐related measures. Future studies should incorporate validated stigma instruments and qualitative assessments of patients’ perspectives on filming to clarify underlying mechanisms and to inform strategies for improving communication and consent processes.

## 5 Conclusions

In conclusion, patients with FMD are more reluctant to be filmed during the neurological examination than patients with other movement disorders. This unwillingness cannot be explained by the higher prevalence of female sex, comorbid psychiatric disorders, or personality styles but may instead be related to FMD‐specific factors. We hypothesize that stigma could play an important role. However, this needs to be investigated in future studies.

## Author Contributions

**Kern Daniela**: conceptualization, data curation, investigation, writing – original draft. **Matic Robert**: investigation, writing – review and editing. **Gattermeyer Kell Lukas**: investigation, writing – review and editing. **Franthal Sebastian**: investigation, writing – review and editing. **Kögl Mariella**: investigation, writing – review and editing. **Katschnig‐Winter Petra**: investigation, writing – review and editing. **Schneller Maria**: data curation, writing – review and editing. **Enzinger Christian**: supervision, writing – review and editing. **Schwingenschuh Petra**: conceptualization, project administration, methodology, supervision, resources, validation, writing – review and editing.

## Funding

The authors have nothing to report.

## Disclosure

Kern Daniela: Travel funding from Ipsen. Matic Robert: none. Gattermeyer‐Kell Lukas: Travel funding from Abbvie, Stada, Merz; Advisory board—Abbvie; Honoraria from Abbvie, Stada. Franthal Sebastian: none. Kögl Mariella: Advisory Boards—Bial, Consultancies—Abbvie, Honoraria from Merz, Abbvie. Katschnig‐Winter Petra: Honoraria from Bial. Schneller Maria: PhD position funded by the Austrian Science Fund. Enzinger Christian: Funding for travel and speaker honoraria from Biogen, Merck, Novartis, Roche. Serving on scientific advisory boards for Novartis, Roche. Schwingenschuh Petra: Funding for travel and speaker honoraria from Abbvie, Bial, Merz. Serving on scientific advisory boards for Abbvie, Bial, Merz, Stada.

## Data Availability

The data that support the findings of this study are available on request from the corresponding author.
